# Global outreach and networking promotion to accelerate tropical diseases elimination

**DOI:** 10.1186/s40249-024-01215-2

**Published:** 2024-06-15

**Authors:** Shan Lv, Xiao-Nong Zhou

**Affiliations:** 1 National Institute of Parasitic Diseases at Chinese Center for Disease Control and Prevention (Chinese Center for Tropical Diseases Research), NHC Key Laboratory of Parasite and Vector Biology, WHO Collaborating Centre for Tropical Diseases, Shanghai, People’s Republic of China; 2 School of Global Health, Chinese Center for Tropical Diseases Research, Shanghai Jiao Tong University School of Medicine, Shanghai, People’s Republic of China; 3 Institute of One Health, Shanghai Jiao Tong University, Shanghai, People’s Republic of China; 4 Hainan Center for Tropical Diseases Research (Hainan Subcenter of Chinese Center for Tropical Diseases Research), Haikou, People’s Republic of China

**Keywords:** Tropical diseases, Cooperation, Networking, Elimination, Sustainable development goals

## Abstract

Cooperation and networking are powerful tools in the combating against tropical diseases. Cooperation on a global scale is essential due to the transboundary nature of tropical diseases. Networking plays a pivotal role in facilitating such cooperation. Both cooperation and networking can foster innovation in disease control programmes. Collaborative research can lead to the development of new drugs and vaccines, while shared surveillance data can enable the early detection and control of disease epidemics. Therefore, consensus of cooperation and networking has been reached during the 7th Symposium on Surveillance-Response Systems Leading to Tropical Diseases Elimination, which reflected in the two documents, i.e., *Consensus for Transboundary Tropical Diseases Control*, and *Action Consensus of the Network of WHO Collaborating Centres Related to NTDs*. These documents will improve the efforts in the fighting against tropical diseases through collective actions to achieve the United Nations’ Sustainable Development Goals (SDGs).

**Graphical Abstract:**

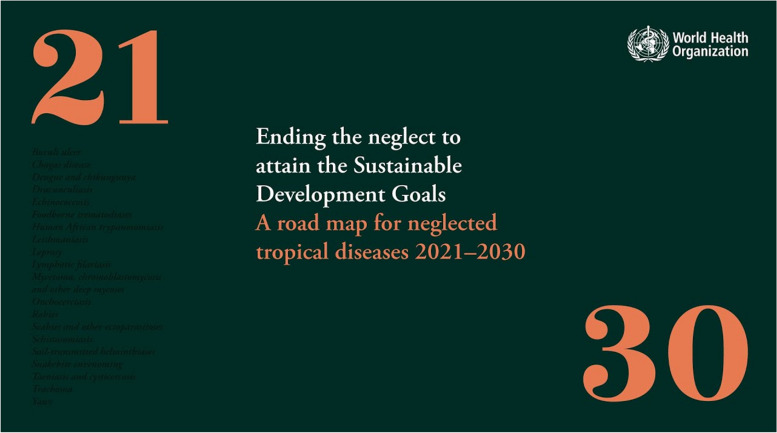

In 2021, the World Health Organization (WHO) launched the second road map for neglected tropical diseases 2021–2030 [1], which has become a global health priority that requires intensive cooperation and networking among various stakeholders. These stakeholders include governments, non-governmental organizations, research institutions, and the private sector, all of whom play critical roles in neglected tropical diseases (NTDs) control strategies. To promote the tropical diseases control strategies, it is necessary to combat tropical diseases safely and effectively in disease endemic countries. With that, surveillance and response systems are the cornerstone that ensure the gathering of information for public health actions to implement global tropical diseases control programmes and achieve the United Nations’ Sustainable Development Goals (SDGs) [2].

Tropical diseases, such as malaria and neglected tropical diseases, including schistosomiasis, leishmaniasis, food-borne trematodiases, cysticercosis and echinococcosis, cause devastating health, social and economic consequences to more than one billion people globally [3, 4]. To accelerate the control and elimination programmes of tropical diseases in endemic areas, cooperation and networking are powerful tools in the combating tropical diseases [5]. Firstly, cooperation on a global scale is essential due to the transboundary nature of tropical diseases. No single country or organization can tackle these diseases alone as they do not recognize national borders. Hence, joint efforts are required to interrupt the spread of these diseases. Secondly, networking plays a pivotal role in facilitating such cooperation. Networks of experts and organizations can pool their resources and expertise to address common challenges. They can also facilitate the exchange of information and best practices, improving capacity building in regions that are most affected by tropical diseases. Thirdly, both cooperation and networking can foster innovation in disease control programmes. Through the exchange of ideas and knowledge, networks can stimulate the development of novel strategies and technologies. These innovations can then be scaled up and disseminated through the network, accelerating the progress towards tropical disease elimination.

However, for cooperation and networking to be effective, challenges that hinder the control and elimination programmes need to be addressed. These include the need for adequate funding, the development of legal and ethical frameworks for data sharing, and the strengthening of health systems in resource-limited settings [6]. Furthermore, cooperation and networking efforts should be inclusive, involving not only experts and policymakers but also affected communities. Their engagement at commnuty level is essential for the design and implementation of culturally appropriate and sustainable interventions. The aforementioned ideas or consensus of cooperation and networking is reached during the 7th Symposium on Surveillance-Response Systems Leading to Tropical Diseases Elimination held in June 2024 in Shanghai cosponsored by the National Institute of Parasitic Diseases at Chinese Center for Disease Control and Prevention, Swiss Tropical and Public Health Institute and WHO, which written in the two important documents, i.e., * Consensus for Transboundary Tropical Diseases Control* (Appendix 1), and *Action Consensus of the Network of WHO Collaborating Centres Related to NTDs* (Appendix 2).

In the * Consensus for Transboundary Tropical Diseases Control*, the significant public health challenges posed by transboundary tropical diseases were well recognized, and the approaches to cooperation, communication and coordination were proposed to be promoted across regions and countries to effectively control the cross-border transmission of tropical diseases, which finally contribute to the global health security, stability and development of local societies. Five consensuses will be taken action among all stakeholders in the near future, including (i) establishing a platform for joint prevention and control of tropical diseases, (ii) promoting information sharing and policy development, (iii) improving capacity building and training, (iv) advancing scientific research and product development, (v) strengthening resource mobilization for coordinated action.

In the *Action Consensus of the Network of WHO Collaborating Centres Related to NTDs*, a group of experts come from WHO Collaborating Centres related to NTDs around the world believed that it is essential to enhance the efficacy of the Network of WHO Collaborating Centres Related to NTDs, which established on the World NTD Day in 2023 (Appendix 3). The network will provide demonstration role of WHO Collaborating Centres dedicated to NTDs control effectively worldwide. Five action consensuses will be executed soon after launching the consensuses, including (i) heightening global awareness of NTDs, (ii) establishing a global exchange platform for NTDs, (iii) strengthening technical and research cooperation for NTDs, (iv) enhancing the evidence-based support capacity, (v) advancing the global elimination of NTDs.

By working together and sharing resources, knowledge, and expertise, efficient cooperation and networking can accelerate the progress towards tropical disease elimination [7]. However, to fully realize this potential, we need to overcome existing challenges and ensure that our efforts are inclusive and equitable. Combating tropical diseases is a shared responsibility, and it is only through collective action that we can hope to achieve our goal.

## Data Availability

The datasets used and/or analyzed during the current study are available from the corresponding author upon reasonable request.

## Appendix 1

### Consensus of Transboundary Tropical Diseases Control

Recognizing the significant public health challenges posed by transboundary tropical diseases, we, the experts in the field of tropical medicine, declare the necessity of coordinated efforts across regions and countries to effectively control the cross-border transmission of tropical diseases through strengthened cooperation, and contribute to the global health security, stability and development.

#### Objectives

1. Strengthen the sharing of information, strategy and policy for unified and coordinated action on transboundary tropical diseases.
2. Enhance transboundary capacity building through training in diagnosis, surveillance, and management of tropical diseases.
3. Boost international collaboration and innovation on R&D to address theoretical, technical, and implementational gaps.

#### Consensuses

1. **Establish a platform for joint prevention and control.** Establish expert committees for history review, experience sharing and updates exchanging, and maintain an open dialogue to address emerging challenges.
2. **Promote information sharing and policy development.** Facilitate the alignment of national policies and develop supportive policy frameworks for joint efforts.
3. **Capacity building and training.** Collaboratively organize trainings to improve monitoring and response capacityies for cross-border disease control.
4. **Promote scientific research and product development.** Jointly conduct scientific research on tropical disease transmission patterns, key technologies, and products among cross-border populations.
5. **Resource mobilization for coordinated action.** Allocate financial and technical resources and seek additional supports from international comnunities for coordinated action.

#### Cooperation priorities

1. **Last mile of tropical disease elimination**. Pool resources and strengths to ensure technical skills and health products needed for the coverage of tropical disease prevention and control reaches the most remote and needy areas.
2. **Cross-border collaboration mechanisms**. Establish and refine cross-border cooperation mechanisms to ensure the swift sharing of information and resources, enhancing overall control efficiency.
3. **Resilient health system construction**. Improve the capability of national and sub-national health systems to respond to public health emergencies related to tropical diseases.

#### Responsibilities of various parties

- Government departments: Responsible for policy coordination, resource allocation, and domestic capacity building, ensuring that national tropical disease control measures align with international standards.
- International organizations: Provide technical support, financial assistance, and capacity building to promote cooperation and coordination among countries.
- Research institutions and universities: Conduct scientific research, provide technical support and training, and drive innovation and R&D.
- Non-Governmental Organizations and Community Organizations: Promote tropical disease prevention and control at the community level, with a special focus on the last mile of health service delivery and outbreak response.

We, the undersigned experts, declare our commitment to the joint prevention and control of transboundary tropical diseases through strengthened cooperation, capacity building, and research promotion.

## Appendix 2

### Action Consensus of the Network of WHO Collaborating Centres Related to NTDs

Neglected tropical diseases (NTDs), a significant public health challenge in the poor populations with limited access to public health and medical resources, are mainly prevalent in tropical and subtropical regions, affecting more than 1 billion people around the world. In response to the United Nations Sustainable Development Goals (SDGs), we believe it is essential to enhance the efficacy of the Network of World Health Organization (WHO) Collaborating Centres Related to NTDs, to utilize the demonstration role of WHO Collaborating Centres dedicated to NTDs and to effectively control of NTDs worldwide, thereby contributing to improve global health and human well-being.

**Network goals:**

1. Strengthen international awareness of NTDs through networked cooperation.
2. Share the latest guidelines and achievements in controlling and eliminating NTDs.
3. The ultimate goal is to support countries to better implement the WHO road map for NTDs, including *A Road Map for Neglected Tropical Diseases 2021 to 2030*, to eliminate NTDs and achieve the SDGs.

**Action consensuses:**

1. **Heighten global awareness of NTDs**. Conduct public health campaigns and policy advocacy to enhance understanding of NTDs, to engage local communities in the planning and implementation of NTDs programs and to garner increased attention from policymakers and decision-makers.
2. **Establish a global exchange platform for NTDs**. Facilitate academic exchanges and sharing of experiences among WHO collaborating centres, develop action plans and set priorities through collaborative deliberation, provide robust support for the prevention and control of NTDs.
3. **Strengthen technical and research cooperation for NTDs**. Deepen technical exchanges, scientific research and innovation. Leverage partnerships to mobilize resources and share expertise. Promote practical cooperation to support regional and global efforts to prevent and control tropical diseases.
4. **Foster capacity building on NTDs**. Develop and disseminate best practices and guidelines for NTDs management. Provide training for healthcare professionals. Strengthen capacity for laboratory testing, diagnostic capabilities, and control management in low resource settings.
5. **Enhance the evidence-based decision-making. **Support local governments in developing and implementing NTDs policies and strategies. Call for strengthening the scope of neglected disease concerns, and propose the updating of the list of NTDs.
6. **Advance the global elimination of NTDs**. Garner support from stakeholders and forge collaborative partnerships. Advocate for increased funding and resources for NTDs programs. Expedite effort towards the elimination of NTDs through coordinated actions and consensuses.

We envision the Network of WHO Collaborating Centres Related to NTDs as a platform for openness, cooperation, and innovation, through effective communication, shared synergies and technological advancements to contribute to ultimately foster a healthier world free from the burden of NTDs.

## Appendix 3

### Network Introduction

WHO encourages its collaborating centres to strengthen communication and collaboration through the establishment of collaborative networks. The establishment of networks of WHO collaborating centres helps to strengthen information exchange with domestic and foreign counterparts and promote technical progress, and to establish partnerships and technical cooperation. Over the past 20 years, more than 10 networks of WHO collaborating centres have been established. On January 30, 2023, the second World NTDs Day, seven WHO collaborating centres active in the field of NTDs, with the support of the WHO, Co-proposed and launched the Network of WHO Collaborating Centres Related to NTDs. The aim of the network is to provide a cooperative platform for all WHO collaborating centres and international organizations working on NTDs to work together to fight against NTDs through enhanced networking. The new network will collaborate in scientific research, communication and training, as well as in WHO-designated missions. The seven initiating centres (in alphabetic order of country) are: the WHO Collaborating Centre for Tropical Diseases (National Institute of Parasitic Diseases of China CDC, China), the WHO Collaborating Centre for Vector Surveillance and Management (National Institute for Communicable Disease Control and Prevention, China CDC, China), the WHO Collaborating Centre for Schistosomiasis Control (Theodor Bilharz Research Institute, Egypt), the WHO Collaborating Centre for Epidemiology, Detection, and Control of Cystic and Alveolar Echinococcosis (in humans and animals) (Istituto Superiore di Sanità, Italy), the WHO Collaborating Centre for Ecology, Taxonomy and Control of Vectors of Malaria, Filariasis, and Dengue (Institute for Medical Research, Malaysia), WHO Collaborating Centre for Epidemiology and Control of Helminth Infections (Swiss Tropical and Public Health Institute, Switzerland), and the WHO Collaborating Centre for Research and Control of Opisthorchiasis (Southeast Asian liver fluke disease) (Khon Kaen University, Thailand).
